# A Challenge for Allergologist: Application of Allergy Diagnostic Methods in Mast Cell Disorders

**DOI:** 10.3390/ijms22031454

**Published:** 2021-02-01

**Authors:** Jan Romantowski, Aleksandra Górska, Marek Niedoszytko, Theo Gulen, Marta Gruchała-Niedoszytko, Bogusław Nedoszytko, Magdalena Lange, Knut Brockow, Michel Arock, Cem Akin, Peter Valent

**Affiliations:** 1 Department of Allergology, Medical University of Gdansk, 80-211 Gdańsk, Poland; klinika.alergologii@gumed.edu.pl (A.G.); mnied@gumed.edu.pl (M.N.); 2 Department of Respiratory Medicine and Allergy, Karolinska University Hospital, 14186 Huddinge, Sweden; theo.gulen@sll.se; 3 Immunology and Allergy Unit, Department of Medicine Solna, Karolinska Institutet, 17177 Stockholm, Sweden; 4 Department of Clinical Nutrition, Medical University of Gdansk, 80-211 Gdańsk, Poland; marta.gruchala-niedoszytko@gumed.edu.pl; 5 Department of Dermatology, Venerology and Allergology, Medical University of Gdansk, 80-211 Gdańsk, Poland; bned@gumed.edu.pl (B.N.); m.lange@gumed.edu.pl (M.L.); 6 Department of Dermatology and Allergy, School of Medicine, Technical University of Munich, D-80802 Munich, Germany; knut.brockow@tum.de; 7 Department of Hematological Biology, Pitié-Salpêtrière Hospital, Pierre et Marie Curie University (UPMC), 75005 Paris, France; michel.arock@aphp.fr; 8 Division of Allergy and Clinical Immunology, University of Michigan, Ann Arbor, MI 48106, USA; cemakin@med.umich.edu; 9 Department of Internal Medicine I, Division of Hematology and Hemostaseology, Medical University of Vienna, 1090 Vienna, Austria; peter.valent@meduniwien.ac.at; 10 Ludwig Boltzmann Institute for Hematology and Oncology, Medical University of Vienna, 1090 Vienna, Austria

**Keywords:** mast cell, anaphylaxis, mastocytosis, hypersensitivity, MCAS

## Abstract

Primary and secondary mast cell activation syndromes (MCAS) can occur in patients with mastocytosis. During the past few years our knowledge about the pathogenesis and disease-triggering mechanisms in MCAS and mastocytosis have increased substantially. Whereas mastocytosis is characterized by an accumulation of neoplastic (clonal) mast cells (MC) in various organ systems, MCAS is defined by a massive and systemic activation of these cells. Mast cells are crucial effector cells in allergic diseases, thus their elevated number and activation can cause severe anaphylactic reactions and MCAS in patients with mastocytosis. However, these cells may also degranulate spontaneously or degranulate in response to non-allergic triggers leading to clinical symptoms. In mastocytosis patients, such symptoms may lead to the diagnosis of a primary MCAS. The diagnosis of a concomitant allergy in mastocytosis patients is challenging. In these patients, a mixed form (primary and secondary) of MCAS may be diagnosed. These patients may also suffer from life-threatening anaphylactic reactions when exposed to allergens. In these cases, the possibility of severe side effects of in vivo provocations can sometimes also limit diagnostic evaluations. In the current article, we discuss the diagnosis and management of patients suffering from mastocytosis and concomitant MCAS, with special emphasis on novel diagnostic tests and management, including allergen microarrays, recombinant allergen analysis, basophil activation tests, optimal prophylaxis, and specific therapies.

## 1. Introduction

Mastocytosis is a group of rare hematologic neoplasms that can be divided into cutaneous mastocytosis (CM) and systemic mastocytosis (SM). In patients with SM, mast cell (MC) accumulations are found in extra-cutaneous organs, such as the bone marrow, spleen, liver, lymph nodes, or the gastrointestinal (GI) tract [1,2,3,4]. A number of different variants of SM have been defined, based on clinical and laboratory variables. These include indolent SM (ISM) and advanced forms of SM, such as aggressive SM (ASM), SM with an associated hematologic neoplasm (SM-AHN) or MC leukemia (MCL) [1,4,5]. The diagnosis of mastocytosis and its variants is established by applying WHO criteria [1,4]. Symptoms in SM may derive from organ damage caused by the local infiltration of neoplastic MC or from the mediators that are produced and released by neoplastic MC [6,7,8]. When the patient (i) is suffering from severe and typical MC mediator-induced symptoms, and (ii) the event is accompanied by a substantial elevation of serum tryptase levels above the individual’s baseline (plus 20% plus 2 ng/mL), and (iii) a significant clinical response to MC-targeting drugs or MC mediator-targeting therapies can be documented, a MC activation syndrome (MCAS) may be diagnosed [2,7,8,9,10,11,12].

MC are well-known effector cells of allergic reactions. In IgE-mediated hypersensitivity reactions allergens cross-link specific IgE molecules that are bound to high affinity FcεRI on MC and thereby trigger degranulation [13,14]. Histamine is one of the clinically relevant mediators of MC that can cause typical symptoms of allergic reactions, such as urticaria, pruritus, rhinitis, bronchospasm, diarrhea, and/or hypotension [13,14,15]. However, other MC mediators, for instance certain leukotrienes or prostaglandins or various chemokines and proteases, may also contribute to the development of these symptoms in allergic reactions [13,14,16]. Apart from IgE-FcεRI interaction and the downstream signaling pathways that are activated by FcεRI cross-linking, MC can also be activated by other mechanisms and receptors expressed on the surface of MC. These include, among others, toll-like receptors, stem cell factor receptor (KIT = CD117), complement receptors, and surface G protein-coupled receptors, including MRGPRX2 [17]. Finally, MC in mastocytosis may be in a “hyperactive” state that may relate to genetic predispositions, such as hereditary alpha tryptasemia (HAT), and may contribute to the severity of allergic reactions. All these mechanisms may act together to cause degranulation of MC and thus lead to the symptoms of anaphylaxis.

In this review, we discuss mechanisms underlying the type and severity of mediator-related hypersensitivity reactions in patients with mastocytosis.

## 2. Hymenoptera Venom-Induced Anaphylaxis in Mastocytosis

It is estimated that about 56–94% of the adult population has been stung by a hymenoptera insect at least once during lifetime, depending on environmental conditions and the geographic region of residence [18]. Clinically relevant, systemic, reactions triggered by hymenoptera stings develop in otherwise healthy adults with a prevalence (percent of affected people at a certain time point) of 0.3–8.9% based on epidemiological studies performed in the last decade [19,20,21]. In patients with mastocytosis, the prevalence is much higher and has been reported to range between 22% and 49% in adults [2,20,22] and between 6% and 9% in children [2,20]. Importantly in patients with SM and insect venom allergy (IVA) the risk of severe anaphylaxis is greater than in patients with IVA without a known MC disorder [21]. The predominant symptoms in these patients are vascular symptoms, including hypotension and syncope [23,24,25]. The association between anaphylaxis and cutaneous mastocytosis (urticaria pigmentosa) has been known for a long time. However, anaphylactic reactions also occur and are quite common in ISM patients with or without skin involvement [21,26]. Moreover, patients with ISM without skin lesions may be at a particular risk of anaphylaxis, with the highest risk of severe sting-induced anaphylaxis reported for male patients with SM without skin lesions and lower basal serum tryptase levels who represent a unique subgroup of ISM with a particularly low MC burden [27]. Many of these patients suffer from bone marrow mastocytosis, a special sub-variant of ISM. In patients with mastocytosis with tryptase levels ranging between 20.4 ug/L and 29.9 ug/L the prevalence of sting-induced anaphylaxis has been reported to be particularly high [28]. However, severe anaphylaxis and MCAS are also seen in patients with a high basal serum tryptase level. Together, anaphylaxis can occur in all forms of mastocytosis and does not typically or more frequently affect patients with advanced SM [23,29].

In the management of patients with anaphylaxis, it is of great importance to establish the correct diagnosis, to avoid triggering factors, and to provide optimal management and treatment. Based on the current guidelines all anaphylactic reactions in the medical history are a clear indication for a detailed allergy diagnosis. It is particularly important in case of insect venom allergy (IVA), as immunotherapy, the only causative treatment, should be initiated in these cases [7,30]. Diagnostic evaluations in patients with mastocytosis may be a clinical challenge. Because of the obvious risk, skin tests with standard insect venom extracts are only recommended in some of the centers. However, most centers recommend screens detecting hymenoptera-specific IgE, which should be performed at least 2–4 weeks after the reaction because of the possibility of a ‘refractory state’ after venom exposure [23,30,31].

Measurements of specific IgE using venom extracts may reveal positive test results for multiple venoms, specific venom allergens, cross-reactive carbohydrates (CCD) or homologous, cross-reacting allergens present in various venoms. Results obtained with full venom extracts may sometimes be negative due to the under-representation of individual molecular allergens in the extract [31]. In several patients with SM, specific serum (s)IgE (detecting allergens) is not detectable probably because in these patients, most of the IgE is fixed to the MC surface membrane. Indeed when these patients undergo MC-reducing therapy, specific IgE may become detectable [32]. It is also worth noting that false-negative skin test results with *Apis mellifera* extract may be due to the lack of certain allergens in the diagnostic and therapeutic extract like Api m10 [33]. Some patients with mastocytosis have negative results in skin tests and in specific IgE detection assays. As mentioned before, this may be due to the fact that most of the IgE is fixed to the surface of MC in these cases. In addition, MC may be in a state of desensitization in SM. In line with this notion, negative results of skin tests and sIgE assays (results below 0.35 kUA/L) are typically seen in patients with sting-induced anaphylaxis and an underlying mastocytosis.

Taking into account the high frequency of severe anaphylactic reactions and reports on fatal sting-induced anaphylaxis [34,35] it is of great importance to use the appropriate sensitivity of sIgE in patients with mastocytosis, which can be improved by lowering the threshold to 0.17 kUA/L without losing good specificity. It is suggested to confirm the IVA in patient with mastocytosis and provide venom immunotherapy (VIT) when skin test results are negative, but sIgE exceeds 0.17 kUA/L [35]. In some patients, it may be possible to determine the presence of sIgE against certain allergens and thus to confirm allergy using functional cellular assays, such as the basophil activation test (BAT) [7]. However, data of the utility of the BAT in patients with sting-induced anaphylaxis with skin tests and sIgE negative results vary according to the center and no robust studies testing this approach in CM or SM are available. In one previous report BAT was not reported to be useful in patients with mastocytosis and the negative results of standard tests [36]. In contrast, in other studies, BAT was found to yield positive results in sensitized mastocytosis patients with a sensitivity ranging between 81% and 87% [23,37].

The treatment of IVA in mastocytosis and MCAS patients is similar to patients without MC disorders. Therapies include allergen immunotherapy, histamine receptor-targeting drugs, and the equipment with an epinephrine emergency kit. Venom immunotherapy (VIT) is the only causative treatment. Although this treatment bears a certain risk of adverse events, the risk-benefit ratio is clearly in favor of VIT and therefore VIT should be administered in all sensitized patients with venom-induced anaphylaxis. In patients with severe anaphylaxis or a high risk to develop severe reactions, including those with mastocytosis and MCAS, VIT should be administered lifelong [7,38]. It is worth noting that in these patients, VIT is also considered to be a relatively safe and effective approach [38,39]. Therefore, recently published guidelines recommend the use of VIT in patients with SM and HVA [21,38,40]. The safety and the effectiveness of VIT were confirmed in patients with mastocytosis although slightly more adverse events were reported compared to patients without MC disorders [21,39]. According to the available reports, the incidence of side effects during VIT in mastocytosis patients ranges from around 18.9% [7] to 34% with the majority of cases (73%) showing reactions in the induction phase and approximately (27%) during the maintenance treatment [25]. These data are similar to the general population of IVA patients treated with VIT [40]. In both cohorts of patients, mastocytosis and non mastocytosis, a majority of the reactions are mild local side effects, and are more frequent to honey bee venom compared to vespid venom. Adrenaline was used to control severe anaphylaxis in 7.6% of the patients with mastocytosis, and 3–7% in non-mastocytosis patients treated with VIT [40] Although the majority of all adverse events in patients with mastocytosis are mild, with local reactions accounting for 56% of these episodes, and 37.5% mild systemic reactions (without respiratory/cardiovascular symptoms), severe anaphylaxis may occur in up to 9% of all cases [25]. Generally vespid VIT was tolerated better (0–15% of side-effects) than honeybee VIT in both groups of mastocytosis and MCAS and non-MC disorder cases [7,41,42]. Monitoring venom-specific IgG4 levels can reflect those patients who achieved tolerance during therapy [25]. Despite a more or less good protective effect in a majority of VIT-treated patients, every patient with mastocytosis with an anaphylactic episode in the history should carry at least 2 self-injectors of epinephrine even when on maintenance treatment with VIT. This recommendation is based on the persistent risk of systemic reactions to insect venoms during VIT [7].

## 3. Inhalant Allergens

Patients with SM may suffer from IgE-dependent allergies against various inhalant allergens and the incidence and prevalence may be the same when compared to patients without a MC disease. In population studies, the frequency of common atopic diseases in patients with mastocytosis ranged between 21% and 31%, depending on the analyzed population [11,13,14,15]. An interesting study was made by Dollner et al. who performed comprehensive rhinologic assessment with specific IgE testing in 11 patients with SM reporting persistent nasal symptoms [43]. Only one of these patients turned out to have a confirmed documented allergy to grass pollen. As mentioned before, such negative test result may not necessarily exclude the presence of an (occult) allergy in all patients. However, it seems as if the prevalence of inhalant allergy in patients with mast cell disorders is similar to that in the general population.

The diagnosis of inhalant allergy is mainly based on skin prick tests and allergen-specific serum IgE. These tools are usually reliable and possess sensitivity and specificity of around 80% [44]. The gold standard–nasal provocation is usually troublesome and difficult to apply routinely in these patients. New in vitro tests, such as the BAT and mast cell activation test (MAT) may also assess cell activation [45,46]. However, no robust validation studies using these assays are available in patients with mastocytosis suffering from concomitant IgE-dependent inhalant allergy.

## 4. Local Anesthetics

In the older literature local anesthetics are sometimes mentioned as potential MC activators through IgE and non-IgE dependent reactions [47]. Up to 3% of cases in the general population experience adverse reactions after local anesthesia [48,49]. Most, if not all, of these reactions are vasovagal in nature, whereas real allergic reactions seem to be rare (<1% of all adverse events). A type IV allergic mechanism appears to be more common than type I mechanism [48]. The vast majority of adverse events result from ‘anxiety’ reactions accompanying the surgical or dentist’s procedure. Another possibility is the intravascular local anesthetic administration that can cause arrhythmias due to suppression of action potential generation in cardiac myocytes [48,49,50]. The fear of MC degranulation induced by local anesthetics origins partly from in vitro studies. Although at low concentration lidocaine decrease basophil and MC degranulation by lowering Ca^2+^ mobilization, at high the same drug may promote MC activation [51,52]. Matito et al. assessed the clinical risk of local anesthetics in patients with mastocytosis [47]. Among 515 administrations, only 4 were symptomatic (less than 1%) and, when specifically reviewing epidural anesthesia, only 2 out of 76 patients had an adverse event (2.5%). Overall, only one case of severe anaphylaxis after local anesthesia was reported in this study [47]. The authors concluded that the risk of allergic reactions and other side effects in patients with mastocytosis is relatively low and acceptable. Nevertheless, prophylactic premedication with anti-mediator-type therapy should be considered in all cases with mastocytosis, and should be applied strictly in all who report previous problems or a previous severe reaction to local anesthesia

## 5. General Anesthetics

The risk of anaphylaxis during general anesthesia in patients with MC disorders is difficult to assess. The incidence of perioperative anaphylaxis in patients with mastocytosis is estimated to be higher with 0.4% compared to that in the general population (0.01%), although the study on patients with mastocytosis was relatively small, may have a selection bias and included only 3 anaphylactic cases in the cohort [47,53]. Most patients undergo surgery without complications, but in some studies anaphylaxis episodes in patients with mastocytosis tend to be more severe than in other patients [54,55,56]. Drugs such as morphine or codeine can degranulate MC in vitro, thus there was a discussion to avoid certain groups of drugs during general anesthesia, including opioids, muscle relaxants, or non-steroid anti-inflammatory drugs [57,58,59,60]. However, this approach might lead to deprivation of proper perioperative analgesia or even needed surgical treatment for SM patients. Furthermore, some authors suggested that severe pain that is not relieved with proper opioid treatment might also cause MC activation [61,62]. Carter et al. reported that the risk of anaphylaxis may be significantly greater in major surgeries, patients with anaphylaxis history and in patients not receiving anti-mediator therapy including histamine receptor (HR)1-targeting and HR2-targeting drug [53]. It has also been reported that patients with mastocytosis who received benzodiazepines as premedication had a lower risk to develop anaphylaxis compared to patients who did not receive benzodiazepines to control stress reactions.

All in all, the use of general anesthesia is not contraindicated in mast cell disorders [53]. It is recommended to perform allergy tests before any procedure only in patients who had allergic reaction to anesthetics or anaphylaxis during anesthesia in the past. In case of inconclusive results, BAT can be performed as an additional test. It is worth noting that BAT has been particularly well validated in healthy population in detection of allergy towards neuromuscular blocking agents [63].

Apart from drugs used in general anesthesia, there is an emerging awareness of possible allergy to antiseptics that are used in perioperative period, particularly chlorhexidine [64]. It is commonly used before surgery and might cause anaphylaxis by IgE-related mechanisms. European Network of Drug Allergy suggests performing a combination of two test methods out of skin prick test, intracutaneous test and serum specific IgE upon patients with suspected chlorhexidine allergy [65]. In case of delayed reactions patch tests are recommended. Additionally, BAT, histamine release test and more recent MAT are being developed as supplementary tests, though they have not yet entered routine application [65,66,67].

Finally, it should be noted that the most important preparative step is to inform the surgery-team and the anesthesiologist that the patient is suffering from an underlying mast cell disorder and from concomitant allergies. In addition, all patients with mastocytosis or MCAS should receive prophylactic HR blockers, including HR1 and HR2-targeting drugs and those who are at high risk should also receive prophylactic glucocorticosteroids before surgery.

## 6. Non-Steroidal Anti-Inflammatory Drugs (NSAID)

Aspirin hypersensitivity is quite prevalent in the general population (0.6–5%) and might generate symptoms of MCA [68,69,70]. These reactions may also aggravate already existing allergic reactions [71]. Taking into account that 9–14% of patients with mastocytosis report severe symptom after taking aspirin or other NSAIDs, there is a reluctance to prescribe NSAIDs in this group of patients [72,73].

The real prevalence of idiosyncratic aspirin hypersensitivity in MC disorders probably does not differ from that in the general population. According to Hermans et al., only 2% of patients with mastocytosis have positive oral provocation test while other studies suggest that 4% of the patients present with hypersensitivity reactions to NSAIDs [74].

Skin lesions in MCA may result from PGD_2_ release [1]. Aspirin is a cyclooxygenase inhibitor that decreases PGD_2_ production in MC and thus might be an effective anti-mediator therapy in mastocytosis. However, the daily dose required to control symptoms of MCA in mastocytosis patients is rather high and may thus lead to severe side effects [75,76,77]. Therefore, aspirin is usually not recommended in patients with mastocytosis, unless the patient did not respond to conventional anti-mediator type drugs.

As a non-IgE-related reaction, the golden standard in NSAID and aspirin hypersensitivity diagnosis remain oral (preferred), intranasal and inhalation provocations [78,79]. Additionally, a few in vitro methods have been developed. These include (1) BAT, (2) sulfidoleucotrienes assay, (3) 15-HETE generation assay [80,81,82]. However, these methods are considered still under development and until now lack proper sensitivity, specificity and validation in larger groups of patients [78,79]. These assays may still be helpful in patients unable to undergo standard provocation protocols as in mastocytosis.

## 7. Food Allergens

Only a very few data on the clinical impact and prevalence of hypersensitivity reactions to food and related allergies in mastocytosis patients are available [2,12,20,83,84].

Over half of the patients with MC disorders report symptoms which are supposedly triggered on consumption of food or beverages [85]. According to Jennings et al., 50.3% of patients with mastocytosis report reactions to food. Another 23.2% reported hypersensitivity to food products. So, in total 73.5% of patients symptoms are associated with diet. Most symptoms have been reported after eating fruits (27%) and vegetables (29%), especially tomatoes and citrus. Other perceived triggers included: dairy products (26%), cereal grains (24%), seafood (24%), food additives (21%) and spices (19%). Interestingly, only 18% of patients indicated hypersensitivity to alcohol, despite the fact that alcohol metabolites have been reported to degranulate MCs directly in vitro, through non-specific activation [86].

In contrast to the numbers above, a recent study by Jarkvist et al. systematically investigated 204 patients with clonal MC disorders and revealed that the prevalence of food hypersensitivity in Swedish mastocytosis patients was only 20.6%; i.e., comparable to the general population [83]. Interestingly, most hypersensitivity symptoms were limited to skin (86%) and/or gastrointestinal tract (45%), as only 2.5% of patients experienced anaphylaxis associated with food products (58). Furthermore, after thorough allergy workup including skin prick tests and specific IgE tests, only 6% of patients turned out to have an IgE-mediated food allergy [83,87]. These observations suggest that food-related symptoms reported by patients with MC disorders mostly result from either a wrong perception of the patients or are due to non-specific causes, whereas the actual prevalence of food allergies may be comparable to that found in the general population [88,89]. Thus, no general elimination diet for mastocytosis patients is recommended without confirmed clinically significant food hypersensitivity [83]. In addition, proper anti-mediator therapy might improve tolerance of certain food products, reducing MCA [1,7].

Microarray–based detection of allergy is a promising method in mastocytosis [32]. The prevalence of chip-positive patients is lower in mastocytosis due to lower levels of total IgE in mastocytosis patients, however the method may be a reliable screening approach for undetected allergies also in patients with mastocytosis [32]. The use of ISAC test in idiopathic anaphylaxis may contribute to the diagnosis in 20% of cases [90].

So far, no specific guidelines were established on food allergy diagnosis in mastocytosis. The recent paper by Vos et al. showed that the cut of level of 0.17 kUA/L sIgE increases sensitivity and specificity of the test in patients with mastocytosis. There is no data on that topic in food allergy jet [35]. Most of the following recommendations concern general population, however based on available publications [11,12,20,83] and our experience, may be used in MCAS and mastocytosis cases.

### 7.1. Nuts

In case of patients reacting to nuts, it is important to distinguish whether they cross-react because of a primary birch pollen allergy or because there is an allergy to a genuine molecular allergen for the food. The most common major allergen in birch pollen, Bet v1, resembles specific allergens in hazelnut (Cor a 1), peanut (Ara h8) and soy (Gly m4). These proteins belong to the PR10 family, they are liable to heat and digestion. In such cases, the reactions are usually mild and limited to local reaction as in oral allergy syndrome. These patients would tolerate food in cooked form. However, if the patients present with anaphylactic reactions, we should analyze sIgE to the genuine food LTP (lipid transfer protein) and storage proteins. LTP allergens, such as in peanut (Ara h9), hazelnut (Cor a 8), walnut and pecan (Jug r3) are stable to heat and digestion. A LTP syndrome is associated with cross-allergy to stone fruits (peach–Pru p3, apple–Mal d3) and with allergy to Artemisia pollens. Storage proteins are main allergens of nuts and markers of primary sensitization. The main recombinant molecular sIgE for peanut (Ara h1, Ara h2, Ara h3), hazelnut (Cor a 9, Cor a 14), walnut and pecan (Jug r1), brazil nut (Ber e1), cashew nut and pistachio (Ana o3) are well-described. These proteins are stable to heat and digestion, associated with systemic reactions to cooked and raw food. Such patients should be consulted by allergologists and dietitians. In case of an anaphylactic reaction in mastocytosis and reaction to LTP and storage proteins the culprit and cross-reactive nuts should be avoided [91].

### 7.2. Fish

Patients with suspected fish allergy require an evaluation with skin prick test and detection of sIgE to most prevalent fish in their diet, such as cod, salmon, carp, tuna and cod parvalbumin (Gad c1—Baltic area, Gad m1—Atlantic area). Other proteins which may lead to anaphylaxis are enolase and aldolase. Parvalbumins are main fish allergens located in small muscles, stable to food processing, and can even become inhalant allergens. Thus, patients with true allergy may develop anaphylaxis due to the inhalation of fish allergen. In cases with negative sIgE to parvalbumin (Gad c1, Gad m1), sIgE to enolase (cod–Gad m2) and aldolase (Gad m3) should be measured. In case of the reaction to other fish i.e., tuna, salmon, hering, megrim, redfish, swordfish specific recombinant IgEs can be measured [91].

### 7.3. Fruits and Vegetables

Allergies to fruits and vegetables are most commonly secondary caused by cross-reaction with pollen allergens (like birch pollen with its main allergen Bet v1) or less commonly true primary food allergy. In case of a clinical reaction, we can apply skin prick tests with commercial extract, native skin prick tests (prick to prick testing), sIgE to the whole allergen or recombinant sIgEs. The last method is a valuable tool in case of nsLTP (type 1) syndrome to fruits (Rosales—apple, peach, apricot, cherry, plum, pear, raspberry, strawberry, mulberry, Ericales—Kiwi, Zingiberales–banana, Sapindales–lemon, tangerine, sweet orange, Vitales—grape [91]. NsLTP protein is found in the tuber of the plant. Patients with positive recombinant sIgE to nsLTP homologues like Pru p3 in peach, Mal d3 in apple should avoid both fresh and processed fruits. In contrast, those reacting primarily to tree pollen cross-reacting proteins like main birch allergen Bet v1 (Mal d1 in apples, Pru p3 in peach) or pollen profilin-related sensitization like birch allergen Bet v2 (peach Pru p3) should eliminate only fresh fruits [91]. Some fruits such as banana, avocado, chestnut, kiwifruit might cross react with latex [92]. The most prevalent vegetables causing anaphylactic reactions are celery, carrot, tomato, bell pepper. Cross reactions with grass pollen (celery) and birch and grass pollen (carrot) are frequent. Main allergens belong to PR-10 and nsLTP protein families [91].

### 7.4. Egg

Anaphylactic reactions are caused by the proteins located in white egg and they can cause inhalant allergy. The main protein–ovomucoid (Gal d1) is heat stable, causing patients to react to all egg-related food. Minor egg molecules like ovoalbumin (Gal d2), transferrin (Gal d3), egg lysozyme (Gal d4) are heat liable, thus patients would react only to raw and slightly heated egg.

### 7.5. Wheat

Allergy to wheat protein is quite common in patients in Northern Europe, and has recently been recognized as an emerging reason for anaphylaxis in adults [84]. Wheat-dependent exercise-induced anaphylaxis (WDEIA) is the common form of omega–5-gliadin hypersensitivity. Severity of reaction depends on the co-factors like exercise, aspirin and alcohol. Specific IgE to Omega-5-gliadin (Tri a 19) is found in 50–80% of allergic patients, being marker of severity and persistence [84,91].

## 8. Hereditary Alpha Tryptasemia

The severity of hypersensitivity reactions may be aggravated by several co-factors. Physical stimuli such as heat, stress, or exercise are commonly described by patients [12,85,93]. In addition, the genetic background may be a clinically relevant co-factor. In particular, a new condition has been described where patients present with (i) elevated basal serum tryptase levels, (ii) additional copies of the alpha tryptase gene (*TPSAB1*) and (iii) a higher risk to develop severe mediator-related symptoms [94,95,96]. This genetic trait found in 5% of the general population and is known as hereditary alpha tryptasemia (HAT) [94,96,97]. Some of these patients present with more severe symptoms of MC activation, including anaphylaxis to hymenoptera stings [95,96]. Hymenoptera venom allergy may also be more prevalent in mastocytosis patients with HAT than in cases without HAT (30.0% vs. 9.9%) [96]. HAT seems to be a predisposing condition and may be particularly relevant when present in patients with IgE-dependent allergies or SM. An interesting aspect is that the prevalence of HAT is particularly high in patients with SM. In fact, HAT is present in about 15–20% of all patients with SM [96]. There is a broad range of symptoms that have been associated with HAT. In a British cohort, the clinical findings were: urticaria/angioedema (51%), abdominal pain (43%), aches/pain (41%), skin flushing (41%), food intolerance (39%), diarrhea (36%), nausea/vomiting (27%), behavioral issues (24%), asthma (20%), eczema (19%), rhinitis (19%), joint hypermobility (17%), dizzy spells (16%), constipation (13%) [97]. However, the prevalence of HAT was similar in patients treated in allergy clinic and in the general population [97]. The diagnosis of HAT may be considered in patients with basal tryptase level is ≥10 ng/mL [95,97]. In some patients with HAT the serum tryptase level is even lower (8–10 ng/mL). Diagnosis of HAT requires genetic analysis using ddPCR described by Lyons and colleagues [98].

## 9. Management of Hypersensitivity Reactions in Patients with Mastocytosis

Many patients with MC disorders present with mediator-related symptoms. These symptoms vary and range from mild and easily tolerable to life-threatening anaphylaxis. As mentioned above it is extremely difficult to distinguish allergic reactions from non-allergic hypersensitivities, taking to account similarity of symptoms and fact that certain triggers might cause both types of mechanisms [1,3,99]. The patients are advised to avoid all possible triggers. However, some triggers cannot be fully avoided (food, the need for anesthesia). Thus, a thorough allergy work-up (see paragraphs above and Table 1) in such cases is required to establish if certain drugs or food products are prohibited and to find a safe alternative. However, still hypersensitivity reaction may occur in these patients. Therefore, there is a consensus that all patients should receive chronic anti-mediator therapy including with HR1 and HR2 blockers and an emergency epinephrine kit [99,100]. Additional drugs might be added depending on organs involved. For example, proton pump inhibitors and cromolyn are considered in patients with gastrointestinal symptoms. Skin involvement might require higher doses of anti-HR1 and topical or systemic glucocorticosteroids. Some of these patients may benefit from high dose (>500 mg daily) of aspirin but the risk of severe side effects and gastrointestinal problems is relatively high in these patients [75,76,77]. In case of anaphylaxis standard treatment with epinephrin should be used followed by glucocorticosteroids and HR1 blockers. Omalizumab is another effective agent that has been suggested for mastocytosis patients suffering from severe IgE-related allergic reactions [101,102,103]. Another potential approach is to apply midostaurin, a KIT and SYK-targeting multi-kinase inhibitor that blocks IgE-dependent mediator release from normal and neoplastic mast cells and basophils in vitro and in vivo [104,105,106]. In addition, midostaurin has been described to suppress mediator-related symptoms and to rapidly increase the quality of life in patients with SM [107]. However, so far, midostaurin can only be prescribed for patients with advanced SM.

Finally, depression and affective disorders usually require psychiatric consultation and administration of antidepressants [7]. Unfortunately, our current pharmacological and diagnostic possibilities are ineffective in about 10–20% of all MCAS patients and in about 10% of all mastocytosis patients [103]. These patients may benefit from the development of new drugs and new therapeutic approaches.

## 10. Conclusions

Patients with MC disorders often present with a variety of mediator-related symptoms that appear either spontaneously or after exposure to certain triggers, such as food, drugs, or insect venoms. The diagnosis of mastocytosis or MCAS in patients suffering from allergy or anaphylaxis may significantly change the management and treatment plan. Therefore, it is crucial to assess the etiology of allergic reactions, including IgE-dependent triggers of anaphylactic reactions as well as aggravating factors and co-morbidities. Diagnostic evaluations in patients suffering from mastocytosis, MCAS and allergic disorders is often a clinical challenge and may require a battery of diagnostic tools and assays, such as IgE and tryptase measurements, hematologic assessments, allergen microarrays, BAT/MAT, or HAT diagnostics. These multidisciplinary evaluations support the physician in arriving at a correct and final diagnosis and in establishing a robust management plan in each individual patient. Such individualized management should may in turn increase safety and the quality of life in patients with mastocytosis and MCAS.

## Figures and Tables

**Table 1:** Test and methods used in hypersensitivity diagnosis in patients with mast cell disorders. SPT—Skin Prick Test; IDT—Intradermal Test; BAT—Basophil Activation Test; MAT—Mast cell Activation Test; NSAIDs—Non steroid anti-inflammatory drugs; AIT—Allergen Immunotherapy [7,23,30,31,32,44,45,46,53,63,64,66,67,79,80,81,82,90,91,108,109].

Allergen Group	Challenge/Provocation	SPT	IDT	sIgE	IgE MiCroarray	BAT	Other/Future Perspectives	Prevalence of Hypersensitivity in Patients with Mast Cell Disorders	Management
Inhalant allergens	+	+	-	+	+	+	MAT, HαT	No data	Symptom medications, AIT
Food allergens	+	+	-	+	+	+	MAT, HαT	20–75%	Avoidance
Hymenoptera venom	− ^1^	+	+	+	+	+	HαT	22–49%	AIT
Local anesthetic	+	+	+	+	−	+		0.7%	Avoidance
General anesthetic	−	+	+	+	−	+	MAT		
Aspirin and NSAIDs	+	−	−	−	−	+	Sulfidoleucotrienes assay, 15-HETE generation assay		Avoidance, desensitization

^1^ In patients with mast cell disorders, insect venom challenge is only recommended during specific immunotherapy to control for therapy efficacy, but not for making the diagnosis.

## Data Availability

No new data were created or analyzed in this study. Data sharing is not applicable to this article.

## Abbreviations

BAT	Basophil activation test
CCD	Cross-reactive carbohydrates
CM	Cutaneous Mastocytosis
ISM	Indolent systemic mastocytosis
IVA	Insect venom allergy
MAT	Mast cell activation test
MC	Mast Cell
MCA	Mast Cell Activation
MCAS	Mast Cell Activation Syndrome
NSAIDs	Non-steroid anti-inflammatory drugs
PBL	Peripheral blood leucocytes
sBTL	Basal serum tryptase level
SM	Systemic Mastocytosis
VIT	Venom immunotherapy
